# Necrological

**Published:** 1880-12

**Authors:** 


### NECROLOGICAL.

"Death is the crown of life:
Were death denied, poor man would live in vain."

The founder of the first Institution in this country for teaching idiots has gone to his rest. Dr. Edward Seguin is no more. Though he has been called from the field of his earthly labors, his noble work survives and will go on conferring its blessings upon a most helpless and dependent class of children and young persons.

Dr. Seguin was a Frenchman by birth, but a most useful and philanthropic American citizen for many years.

Peace to his honored ashes.

A good and true man, and an excellent dentist, has been called to his home, in Robert Arthur, M. D., D. D. S., of this city. The cause of his death was Gangrene, superinduced by embolic occlusion of the main arteries of his leg. He filled several important and honorable positions in Dentistry, as Professor, &c., in several Colleges. He was a man of culture and contributed quite frequently to the Journals of the Profession.

